# Composition of Minerals and Volatile Organic Components of Non-Centrifugal Cane Sugars from Japan and ASEAN Countries

**DOI:** 10.3390/foods12071406

**Published:** 2023-03-26

**Authors:** Fitriyono Ayustaningwarno, Yonathan Asikin, Ryo Amano, Nam Tuan Vu, Siti Hajar-Azhari, Gemala Anjani, Kensaku Takara, Koji Wada

**Affiliations:** 1 Nutrition Science Department, Faculty of Medicine, Diponegoro University, Semarang 50275, Indonesia; 2 Center of Nutrition Research (CENURE), Diponegoro University, Semarang 50275, Indonesia; 3 Department of Bioscience and Biotechnology, Faculty of Agriculture, University of the Ryukyus, Okinawa 903-0213, Japan; 4 United Graduate School of Agricultural Sciences, Kagoshima University, Kagoshima 890-0065, Japan; 5 Institute of Genome Research, Vietnam Academy of Science and Technology, Hanoi 122100, Vietnam; 6 Department of Food Science, Faculty of Food Science and Technology, Universiti Putra Malaysia, Selangor 43400, Malaysia

**Keywords:** non-centrifugal cane sugar, mineral, volatile organic component, MS-e-nose, Maillard reaction product, production origin

## Abstract

Non-centrifugal cane sugar (NCS) is an unrefined dehydrated form of sugar syrup produced worldwide. To date, there is a lack of differentiation in the key nutrients and flavor qualities of NCS products among countries, which makes it difficult for interested parties to select NCSs suitable for their needs. This study aimed to evaluate the minerals and volatile organic components (VOCs) in NCS products from Japan and ASEAN countries. Mineral components were determined using inductively coupled plasma atomic emission spectroscopy (ICP-AES). VOCs and their aroma profiles were examined using gas chromatography–mass spectrophotometry (GC-MS) and MS-e-nose analyses, respectively. The total minerals content in Japanese NCSs ranged from 228.58 to 1347.53 mg/100 g, comprising K, Ca, Mg, P, and Na (69.1, 16.6, 7.9, 4.5, and 3.2%, respectively); their average total amounts were as high as those of Malaysia and Indonesia origins (962.87, 984.67, and 928.47 mg/100 g, respectively). Forty-four VOCs were identified, of which concentrations of pyrazines, furans, and pyranones varied significantly among the NCSs. Additionally, the MS-e-nose analysis provided a multivariate differentiation profile of the NCS products based on differences in the intensities of the VOC ion masses. Nine statistical clusters were presented, wherein certain NCS products of ASEAN origin had volatile profiles comparable to those of the Japanese products. These outcomes suggest that the origin of production greatly influences the mineral and VOC compositions of NCS, affecting their quality traits.

## 1. Introduction

Unrefined noncentrifugal sugar is produced from various sugar sources such as plant carbohydrate reserves, including sugarcane, beets, and palms [1,2]. Unlike refined sugar, non-centrifugal cane sugar (NCS) is manufactured from sugarcane sugar syrup without molasse removal. Thus, it has distinct physicochemical traits such as brown color, moisture content, and water activity. Furthermore, it is rich in minerals, volatile organic components (VOCs), and various bioactive compounds that exhibit biological benefits such as antioxidant, anti-inflammatory, anti-obesity, and antidiabetic properties [3]. NCS is known in many countries and regions by different names such as kokuto and kurozato (Japan), duong mat mia (Vietnam), gula melaka (Malaysia), gula merah (Indonesia), gur and jaggery (South Asia), and panela (South America) [4]. NCS products are widely used in various applications, such as table sugar, snacks, and ingredients for a variety of other foods and drinks [5]. Generally, NCS is recognized as safe. Although some studies have mentioned the possibility of the generation of acrylamide and polycyclic aromatic hydrocarbons in NCSs, the amounts are tolerable in the human metabolic system [3].

The quality of NCS products is determined by their nutrient and flavor properties [1,4,6]. With the growing global interest in health foods, NCS has recently gained increasing attention from health-concerned consumers, and interested parties are seeking suitable quality attributes of NCSs to meet their needs [7]. Among these traits, the key nutrients and flavor components are minerals and VOCs [8,9]. Minerals are important nutrients that maintain many functions in human organs; additionally, they affect the taste characteristics of NCS, such as bitterness [8,10]. The slow evaporation process during NCS production is responsible for non-enzymatic browning reactions, which generate various Maillard reaction products (MRPs) in NCS [6,11]. Most of these substances emit the desired aroma characteristics that can enhance the overall quality of NCS products, such as the roasted peanut odor from pyrazines and sweet cotton candy from furanones [8,11,12]. NCSs vary in minerals and VOCs for several reasons such as their production origin and processing methods [8,13]. In our previous studies, we confirmed the importance of minerals and VOCs in determining the NCS quality [1,8,11,14].

The use of electronic sensing to evaluate the aroma profile of food materials has become popular for differentiating samples owing to its promising discrimination power [8,15,16]. The electronic nose (e-nose) has been used in food quality studies to distinguish materials with different aroma characteristics and intensities [15,16]. Although the human nose can be used to evaluate aromas, the judgments can be biased and have detection limits [17]. Analytical instruments such as gas chromatography–mass spectrometry (GC-MS) have also been used to measure VOCs; however, GC-MS is time-consuming and occasionally requires complicated sample preparation [18]. E-noses have been developed using various types of sensors, including metal oxide semiconductors, conducting polymers, acoustic waves, and MS [17,18]. An MS-based e-nose offers a fast and non-destructive alternative for measuring VOCs [8,18]. This analytical method has been successfully applied to differentiate Japanese NCS from different islands and unrefined sugars from various plant sources [1,8].

To date, the compositional analysis of NCS has been limited to domestic products in countries such as Japan, Brazil, India, Mexico, and Malaysia [8,19,20,21,22]. Moreover, there is a lack of assessment of nutrient and flavor components in NCSs across countries. Because adequate comparisons between quality attributes from different countries are not available, interested parties and consumers with specific quality requirements could have difficulty selecting suitable NCSs, particularly for products from Japan and developing nations, such as ASEAN countries. To the best of our knowledge, this is the first study to evaluate the key nutrients and flavor qualities of NCS products from Japan and other ASEAN countries. This study primarily aimed to discriminate between minerals and VOCs in NCSs from Japan and ASEAN countries. The mineral composition of the NCS products was analyzed using inductively coupled plasma–atomic emission spectroscopy (ICP-AES), whereas the VOCs were analyzed using GC-MS. Additionally, aroma profiles were evaluated using the MS-e-nose method and chemometric data analysis. These three analytical instrumentation approaches were applied to reveal the key quality attributes of NCS products, which are considered the primary measures for NCS selection by industrial users and household consumers.

## 2. Materials and Methods

### 2.1. Sample Preparation

**Figure 1:**
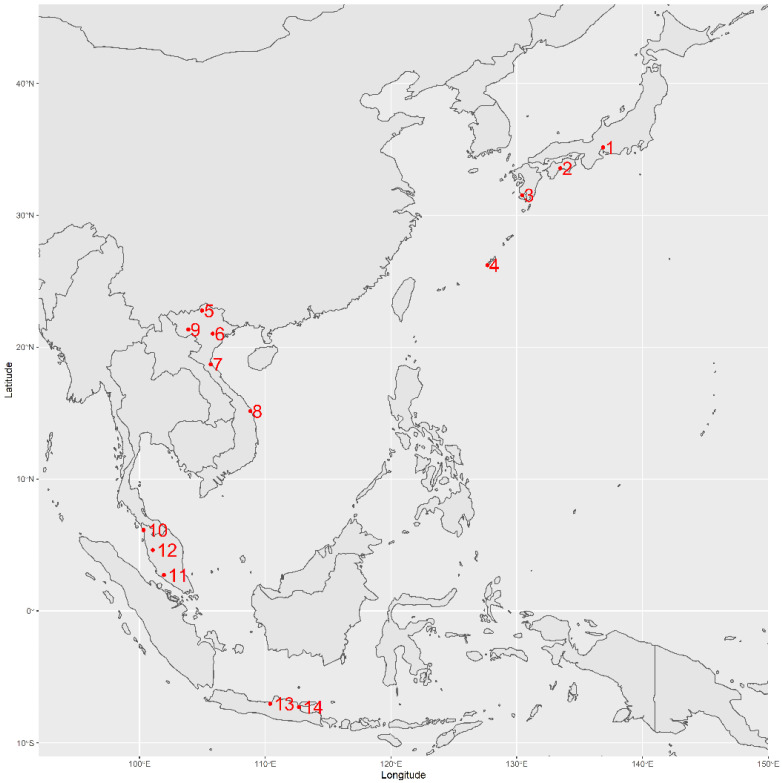
Non-centrifugal cane sugar (NCS) product source distribution in Japan and ASEAN origins. Dots on the map represent the city of origin for each NCS sample. Numbers represent the country and city name. 1. JP-Aichi, 2. JP-Kochi, 3. JP-Kagoshima, 4. JP-Okinawa, 5. VN-Ha Giang, 6. VN-Hanoi, 7. VN-Nghe An, 8. VN-Quang Ngai, 9. VN-Son La, 10. MY-Kedah, 11. MY-N. Sembilan, 12. MY-Perak, 13. ID-Central Java, 14. ID-East Java.

NCS products comprising 20 samples were obtained from Japan and three ASEAN countries: ten NCSs were obtained from Japan, representing four prefectures (Aichi, Kochi, Kagoshima, and Okinawa); five from Vietnam (Ha Giang, Hanoi, Nghe An, Quang Ngai, and Son La); three from Malaysia (Kedah, Negeri Sembilan, and Perak); and two from Indonesia (Central Java and East Java). The NCS products were obtained from sugarcane-producing areas and selected based on popularity and availability, as informed by local retailers or producers. The locations of the NCS sources are shown in Figure 1. All NCS products were procured during the local production session in 2020, arrived at the laboratory within one week in a sealed container, and were stored at −30 °C prior to analysis.

### 2.2. Standards and Reagents

Mineral (multi-element) standard solutions, 1,2-dichlorobenzene-D4, and alkanes (C_7_–C_30_) were purchased from Sigma-Aldrich (St. Louis, MO, USA). Phosphorus standard solution and nitric acid were obtained from Fujifilm Wako Pure Chemical Industries (Osaka, Japan). All other reagents were of analytical grade.

### 2.3. Mineral Composition Analysis

The mineral compositions of the NCSs were determined using ICP-AES [8]. Briefly, NCS (0.25 g) and nitric acid (4 mL) were placed in a sealed decomposition vessel (SAN-AI Kagaku Co., Ltd., Nagoya, Japan) and heated in a forced-convection oven at 160 °C for 8 h. The mixture was dissolved in 50 mL distilled water, filtered, and injected into a Shimadzu ICPE-9000 ICP-AES system (Shimadzu Corporation, Kyoto, Japan). The carrier gas was argon, and the flow rates of the carrier and auxiliary gases were 0.7 and 0.6 L/min, respectively. The plasma exposure time was 30 s, at a flow rate of 10 L/min. Mineral concentrations were calculated using a calibration curve for each mineral standard and expressed as mg/100 g. All analyses were performed in triplicate.

### 2.4. Volatile Composition Analysis

The VOCs in the NCSs were extracted using solid-phase microextraction (SPME) and analyzed using GC-MS [14]. Briefly, 3 g of NCS and 20 µL of 1,2-dichlorobenzene-D4 (2.5 μg/mL in MeOH) as an internal standard were placed in a closed 20 mL vial and heated at 60 °C for 5 min. Volatile compounds were then extracted using SPME fiber containing divinylbenzene/carboxen/polydimethylsiloxane (Supelco Inc., Bellefonte, PA, USA) whilst heated at 60 °C for 15 min. The divinylbenzene/carboxen/polydimethylsiloxane fiber was selected for its ability to extract volatile flavor substances from different chemical classes, including low-molecular-weight compounds and polar volatiles. The GC-MS analysis was performed using a Shimadzu GC2010-QP2010 PLUS (Shimadzu Corporation, Kyoto, Japan), equipped with a Shimadzu AOC-5000 Plus CTC PAL GC Autosampler and DB-WAX column (30 m × 0.25 mm, 0.25 μm) (Agilent J&W, Santa Clara, CA, USA), and helium was used as a gas carrier with a flow rate of 35 cm/s. The GC injector temperature was set at 250 °C. The SPME fibers were injected at a split ratio of 1:2. The oven temperature was set initially to 55 °C, increased to 220 °C at a rate of 6 °C/min, and held isothermally at 220 °C for 2.5 min. For MS detection, the electron impact ion source and interface temperatures were set to 230 °C, interface temperatures were set to 200 °C, and electron ionization was performed at 0.7 kV. The mass acquisition scan range and rate were (*m*/*z*) 33–450 amu and 1.77 scans/s, respectively. Volatile compounds were identified by MS similarity (FFNSC and NIST08 libraries) and based on their linear retention indices (RIs) using a homologous series of *n*-alkanes (C_7_–C_30_). The weight intensity of the peak was calibrated to the MS response of the internal standard, and the content of the volatile compounds was expressed as µg/100 g. The relative percentage (%) of each compound was determined by measuring the peak area response to the total number of detected peaks, and the relative percentages of the compounds with the same chemical group were summed. All analyses were performed in triplicates.

### 2.5. MS-e-nose Analysis

This MS-e-nose approach allows for non-targeted volatile profiling without chromatographic peak separation requirements and provides digital fingerprints from the acquired mass spectra of the volatiles. The MS-e-nose profiles of the NCSs were analyzed using a GERSTEL ChemSensor (GERSTEL, Mülheim, Germany) on an Agilent 7890A-GC-5975C MS system equipped with an Agilent G1888 HSS autosampler (Agilent J&W) [1,8]. Briefly, 3 g of NCS was placed in a 20 mL vial, and the headspace was extracted at 80 °C for 10 min and then pressurized into the GC injection port at 11 psi for 0.3 min The injection temperature was set at 250 °C, and the injection split ratio was 1:10. The sample and transfer lines were set at 170 and 210 °C, respectively. The GC was equipped with an HP5-MS column (15 m × 0.25 mm, 0.25 μm, Agilent J&W), with helium as the gas carrier. The oven temperature was programmed to 40 °C for 0.5 min and then increased to 200 °C at a rate of 60 °C/min, and held isothermally at 200 °C for 1.5 min. The total duration of GC analysis was 4.67 min. The MS ion source and interface temperatures were programmed at 250 °C, and the electron ionization was set to 70 eV. The volatile components were scanned at *m*/*z* 30–290. The total mass spectral intensities of the scanned ion masses of the volatiles were converted into chemometric datasets. All analyses were performed in triplicates.

### 2.6. Statistical Analysis

A multivariate visual graph of VOCs in the NCSs was constructed using principal component analysis (PCA) in a mean-centered structure (JMP, SAS Institute, Cary, NC, USA). Ion masses of total volatiles from the MS-e-nose analysis were also statistically computed through PCA and hierarchical cluster analysis (HCA) plots using Pirouette 4.5 (Infometrix, Bothell, WA, USA). Both multivariate computations of the MS-e-nose analysis were based on mean-centered preprocessing data, and the HCA plot was obtained using Euclidean distance and incremental linking. Clusters were obtained using a 0.900 similarity index.

## 3. Results and Discussion

### 3.1. Mineral Composition

**Figure 2:**
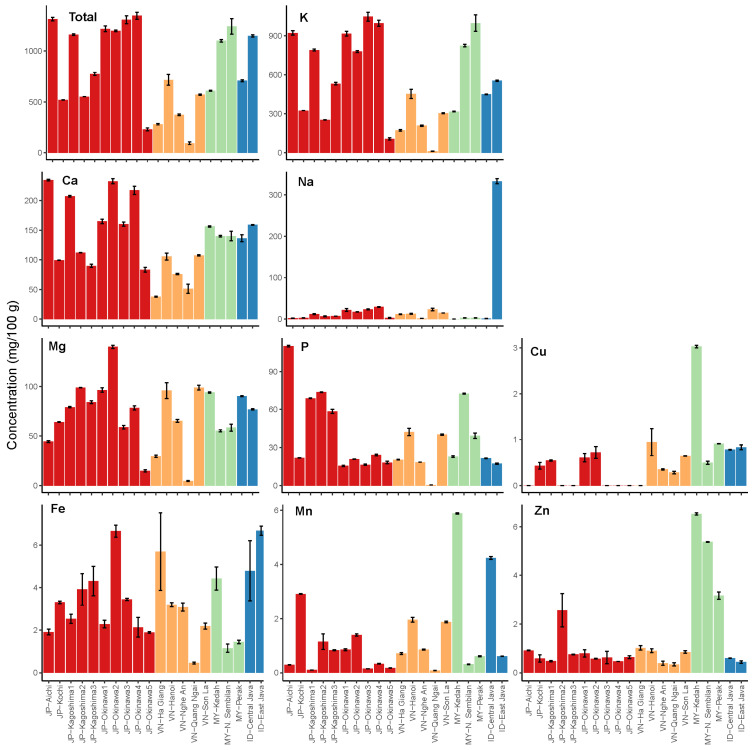
Mineral composition of NCS products from Japan and ASEAN countries (Vietnam, Malaysia, and Indonesia). Each value is expressed as the mean ± standard deviation of three replicates. Statistical significance analysis of the total mineral content is presented in the Supplementary Material, Table S1.

NCS products from Japan and ASEAN countries (Vietnam, Malaysia, and Indonesia) contained major elements (K, Ca, Na, Mg, and P) and microelements (Cu, Fe, Mn, and Zn) (Figure 2). The total mineral contents ranged from 228.58 to 1347.53; 92.46 to 716.13; 609.91 to 1243.01; and 708.83 to 1148.12 mg/100 g for Japan, Vietnam, Malaysia, and Indonesia, respectively. Among the evaluated NCS products, the highest total mineral content in each country was obtained from JP-Okinawa4, VN-Hanoi, MY-Perak, and ID-East Java. These NCS products had lower mineral contents than those previously reported for unrefined brown sugars from Japan and Pakistan, which ranged from 1648 to 2972 and 1453 to 2153 mg/100 g, respectively [8,23].

Notably, the VN-Quang Ngai and JP-Okinawa5 NCS products contained significantly lower total mineral content than the others. These products are suspected to be blended NCSs, most likely prepared not only from fresh sugarcane juice but also probably mixed with other adulterants such as reused NCSs, refined sugars, and molasses. The suspected blended Japanese NCS, JP-Okinawa5, was prepared by mixing freshly prepared sugarcane juice with refined sugar and molasses. In Japan, this product is known as processed brown sugar or kako-kokuto [24]. Because refined sugar has a significantly lower total mineral content than unrefined brown sugar [6], JP-Okinawa5 had a lower total mineral content than the other Japanese NCS products. Chen et al. [25] also reported the production of NCS products made from a mixture of refined sugar and molasses at a lower commercial price compared with regular NCS product, which is an unrefined brown sugar made from sugarcane juice via thermal processing. However, as unrefined NCS from sugarcane juice is perceived to be healthier by consumers, the price gap can be used to represent this refined sugar-mixed NCS as unrefined.

The mineral compositions of NCS products of Japanese and ASEAN origin, which are predominantly composed of K, Ca, Na, Mg, and P, are in agreement with our previous study [8]. The potassium content of NCSs ranged from 97.80 to 1076.00, 11.00 to 492.00; 1.02 to 492.00; 314.00 to 1052.00; and 446.00 to 558.00 mg/100 g for Japan, Vietnam, Malaysia, and Indonesia, respectively. The highest K content in each country was in JP-Okinawa3, VN-Hanoi, MY-Perak, and ID-East Java. Potassium was the most abundant mineral in the NCS as part of the sugarcane itself or plants in general. Plants require K to regulate the uptake, transport, and use of water and other nutrients [26]. Calcium was the next most abundant mineral in NCS after potassium. Calcium content ranged from 79.00 to 236.00, 37.2 to 112.00, 131.20 to 157.20, and 130.20 to 159.80 mg/100 g for Japan, Vietnam, Malaysia, and Indonesia respectively. The highest Ca content was obtained in JP-Okinawa2, VN-Hanoi, MY-Kedah, and ID-East Java. Calcium is widely used during liming in NCS production to suppress sucrose inversion and to test whether calcium reacts with phosphorus to form calcium phosphate, which acts as a flocculant. Calcium phosphate can adsorb phenols, proteins, gummy matter, and waxes, which are known impurities in sugarcane juice [22]. Calcium hydroxide can also be used to remove impurities from NCS [3].

Natrium content ranged from 1.81 to 30.40, 1.33 to 26.80, 0.03 to 3.28, and 1.15 to 338.00 mg/100 g for Japan, Vietnam, Malaysia, and Indonesia, respectively. The highest Na content was observed in JP-Aichi, VN-Quang Ngai, MY-N. Sembilan, and ID-East Java. Although most of the NCS products contained less than 30.5 mg/100 g of natrium, a significant amount of natrium (more than 10-fold) was found in the NCS from ID-East Java. This exception can be explained by the use of sodium metabisulfite during NCS production. In Indonesia, this method has been reported to increase the yield and total dissolved solids in produce [27]. Sodium metabisulfite is also commonly added at 0.1% to increase the shelf life [28]. In addition to this chemical, sodium bisulfite is also used in India as an NCS preservative [22].

Magnesium content ranged from 13.86 to 141.40; 4.30 to 104.80; 54.40 to 90.80; and 76.40 to 91.00 mg/100 g in Japan, Vietnam, Malaysia, and Indonesia, respectively. The highest Mg content in each country was JP-Okinawa2, VN-Hanoi, MY-Kedah, and ID-Central Java [29]. Mg is the central element in plant chlorophyll and is essential for photosynthesis and sugar production in sugarcane plants [30]. Furthermore, P content ranged from 15.08 to 110.60, 0.47 to 45.40, 22.20 to 73.00, and 17.04 to 21.80 mg/100 g for Japan, Vietnam, Malaysia, and Indonesia, respectively. JP-Aichi, VN-Hanoi, MY-N. Sembilan, and ID-Central Java had the highest P contents. Phosphorus has been used as a fertilizer to increase sugarcane yield [31] and as an additive in the liming process in the form of calcium phosphate to reduce impurities in NCS [22].

Regarding microelements, NCS products from Malaysia contained high Zn levels that ranged from 3.02 to 6.58 mg/100 g. Zinc addition during sugarcane planting has been reported to increase the concentrations of other minerals, such as Mn, in the whole plant [32]. This might be triggered by a higher Zn supply, which causes an increase in the Mn concentration in the roots and slight alterations in the leaves and stalks. High Zn and Mn contents were found in MY-Kedah but not in other Malaysian NCS products. Moreover, Jain et al. [32] also mentioned that high Zn concentrations might be responsible for reducing the Fe content, which was observed in NCS products from MY-N. Sembilan and MY-Perak.

Minerals are important micronutrients in human diet. Examples of important minimum mineral dietary reference intakes (DRI) are as follows: potassium, with a minimum DRI for adult males of 4.5 g/day, maintaining cell fluid volume and normal cell function. Calcium, with a minimum DRI for adult males of 800 mg/day, is one of the most important components of bones and teeth and is involved in blood coagulation and intercellular communication. Furthermore, magnesium, with a minimum DRI for adult males of 200 mg/day, is a building block for bones and teeth, and is involved in muscle relaxation [10]. A 2200-calorie diet for healthy adult men and an 1800-calorie diet for healthy adult women allows for the consumption of 36 and 20 g of sugar, respectively [33]. Combined with the DRI of minerals, the use of NCS products of Japanese and ASEAN origin as substitutes for refined sugar would increase the intake of K, Ca, and Mg by approximately 4.4, 6.1, and 12.9% for men, and 2.4, 3.4, and 7.2% for women, respectively.

Most major mineral substances in food are associated with bitterness, and Na is responsible for salty taste [9]. The mineral amounts in NCS products may influence taste perception, particularly bitter tastes [8,34]. Among these minerals, Mg is strongly associated with bitterness [29]. These minerals, along with sugar, amino acids, and phenolic components, impart rich taste properties to NCS [8,35]. Moreover, the mineral components of NCS can positively affect the overall sensory attributes of foods and beverages when used as sweet-source ingredients compared to other sugar products [36,37]. Compositional variation in the minerals of NCS products from Japan and ASEAN countries not only impacts their taste characteristics, but also alters their practical applications as sugary ingredients. These outcomes can thus provide information on not only the nutritional composition of mineral substances but also the possible taste attributes of these unrefined sugars for health-concerned consumers [7].

### 3.2. Volatile Organic Components (VOCs)

**Figure 3:**
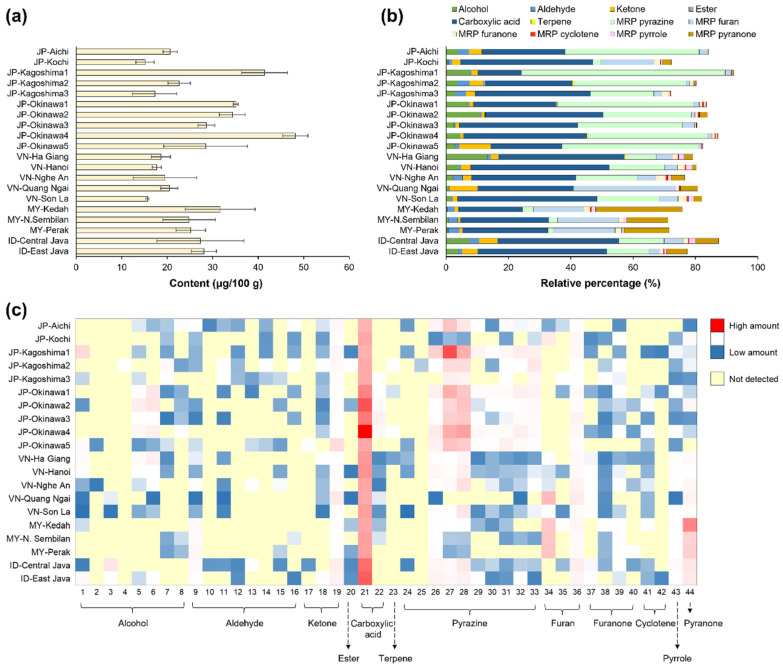
(**a**) Total content, (**b**) relative percentage, and (**c**) heatmap visualization of volatile organic components (VOCs) of NCS products from Japan and ASEAN countries (the compound numbers in the heatmap refer to the volatile organic components in Supplementary Material, Table S2).

The composition of VOCs in NCSs can determine their aroma traits because they can be detected by olfactory receptors [38]. NCS products from Japan and ASEAN countries have different VOC contents and compositions based on their origins, wherein 44 compounds were detected, as shown in Figure 3. The total content of VOCs in Japanese, Vietnamese, Malaysian, and Indonesian NCS products ranged from 12.94 to 51.02, 14.35 to 27.43, 20.61 to 39.62, and 19.80 to 38.08 µg/100 g, respectively (Figure 3a). The highest total VOCs in each country were JP-Okinawa4, VN-Nghe An, MY-Kedah, and ID-Central Java, indicating their high potency. Besides JP-Okinawa4, the other Japanese NCS products with greater VOC content were obtained in JP-Kagoshima1, JP-Okinawa1, and JP-Okinawa2. In contrast, JP-Kochi had the lowest total VOCs content, followed by JP-Kagoshima3 and VN-Son La, indicating their low aroma strength, which could influence the overall sensory properties of NCS products [8,39]. Interestingly, although the correlation between VOCs and mineral content in the evaluated NCS products was moderately positive (Pearson correlation coefficient, r = 0.5892), the contents of these key flavor quality traits in the NCS from VN-Quang Ngai were consistently lower than those of other products (Figure 2). This indicates that the suspected Vietnamese blended product not only has low nutrient attributes but also weaker odor strength.

Generally, the notable VOCs of the NCS products include alcohols, aldehydes, ketones, esters, carboxylic acids, terpenes, and MRPs, such as pyrazine, furan, furanone, cyclotene, pyrrole, and pyranone (Figure 3b) [1,8,14]. The proportion of the relative concentrations of the chemical groups of the VOCs represents the chemical complexity related to the aroma diversity in the NCS. The median number of identified VOC was 26 compounds, of which the greatest number of VOCs (30 compounds) was observed in the NCS from ID-Central Java, and the least identified NCS was MY-Perak, with 17 compounds (Figure 3c). Moreover, most NCS products contain all the notable volatile chemical groups. However, some substances, such as esters, terpenes, cyclotenes, pyrroles, and pyranones were not detected in particular NCS products, suggesting that variations in VOCs could represent the complexity of NCS flavors of different origins [8,12].

**Figure 4:**
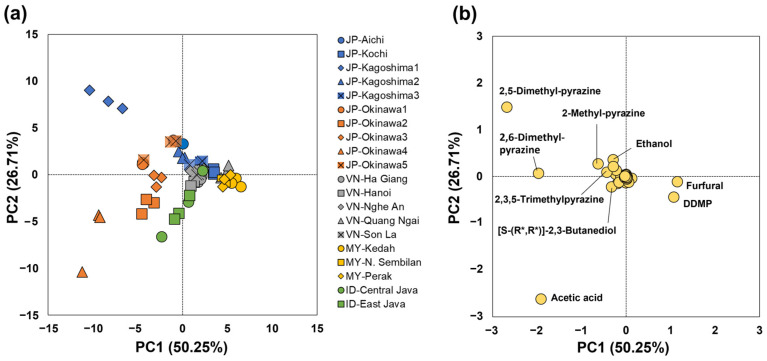
(**a**) PCA scores and (**b**) factor loadings of volatile organic components (VOCs) of NCS products from Japan and ASEAN countries.

The relative percentages of predominant carboxylic acids ranged from 13.84 to 42.52%, 30.80 to 44.68%, 20.54 to 28.43%, and 38.80 to 41.42% for Japanese, Vietnamese, Malaysian, and Indonesian NCS products, respectively (Figure 3b). The NCSs with the highest proportion of carboxylic acids in each country were JP-Kochi, VN-Son-La, MY-N. Sembilan, and ID-East Java. This group comprises a major carboxylic acid, acetic acid, and a small amount of butanoic acid (Figure 3c; Supplementary Table S2). The NCS from JP-Okinawa4 contained the highest amount of acetic acid, followed by JP-Okinawa2 and ID-East Java. Acetic acid, which may provide a pungent odor [40], is present in NCS because of microbiological fermentation occurring during the lag time in raw cane juice storage before the evaporation process [41]. This compositional outcome was also confirmed through a PCA plot, wherein JP-Okinawa4 was distinct from other NCS products owing to the high concentration of acetic acid. This Okinawan specialty NCS plotted in the negative direction of the first two PCs of the score plot, as well as for acetic acid in the loading plot (Figure 4a,b).

Furthermore, NCS from JP-Okinawa4 contained the highest amount of MRP components, followed by those from JP-Kagoshima1 and JP-Okinawa1. Conversely, JP-Kochi had the lowest MRP concentration, followed by JP-Kagoshima3, and VN-Son-La (Figure 3b). This result is also in agreement with the mineral content of the NCS products (Figure 2), suggesting that NCS with lower nutritional content may also lack various precursors for generating key aroma compounds, such as MRPs. However, an NCS with high key nutrients, such as minerals, could also potently contain pleasant aroma characteristics from volatile MRPs, as these volatiles are associated with sweet and roasted aromas that are likely desirable in the NCS [8,11,14].

The relative percentage of MRP pyrazines ranged from 2.47 to 65.47, 0.19 to 19.91, 1.61 to 3.33, and 13.39 to 14.53% for Japanese, Vietnamese, Malaysian, and Indonesian NCS products, respectively (Figure 3b). The NCS with the highest proportions of pyrazines were JP-Kagoshima1, VN-Son La, MY-Kedah, and ID-Central Java. All Japanese NCS products, except JP-Kochi, possessed a higher MRP pyrazine content than NCSs produced in ASEAN countries. With the second highest total volatile content, JP-Kagoshima1 was composed of 65.5% pyrazines (Figure 3a,b), mainly 2,5-dimethyl-pyrazine (2,5-DMP) with 12.8 µg/100 g and 2,6-dimethyl-pyrazine (2,6-DMP) with 5.9 µg/100 g (Figure 3c; Supplementary Table S2). 2,5-DMP can occur during Strecker degradation of glucose, causing distinctive nutty and roasted odors, whereas 2,6-DMP provides nutty and sweet aromas [11,42]. Moreover, PCA plots confirmed the differentiation of Japanese NCS products from those containing higher concentrations of 2,5-DMP and 2,6-DMP (Figure 4). Therefore, these two pyrazines can be considered potential chemical markers of NCS products from Japan.

The relative percentages of MRP pyranone ranged from 0.00 to 2.98%, 1.29 to 5.37%, 13.29 to 27.76%, and 6.60 to 7.44% for the Japanese, Vietnamese, Malaysian, and Indonesian NCS products, respectively (Figure 3b). The NCSs with the highest proportions of pyranone in each country were JP-Kochi, VN-Quang Ngai, MY-Kedah, and ID-Central Java. The only compound in this group was 2,3-dihydro-3,5-dihydroxy-6-methyl-4H-pyran-4-one (DDMP), which was detected in high levels in Malaysian and Indonesian NCS products. Furthermore, the PCA plot shows the importance of DDMP in the Malaysian NCS (Figure 4). DDMP is produced during the intermediate stage of the Maillard reaction and is primarily a bitter compound [43]. Meanwhile, Cutzach et al. [44] reported that DDMP could be produced in large amounts during the heating of a glucose–proline mixture, producing a toasty caramel aroma.

MRP furan group relative percentages were measured between 1.03 and 17.16%; 4.10 and 32.58%; 16.28 and 19.93%; and 3.45 and 6.06% for Japanese, Vietnamese, Malaysian, and Indonesian NCS products, respectively (Figure 3b). The NCSs with the highest proportion of furans in each country were JP-Kochi, VN-Quang Ngai, MY-Perak, and ID-Central Java. All Japanese NCS products, except JP-Kochi, contained fewer furans than the NCS products from other countries. VN-Quang Ngai contained the highest proportion of furans (32.58%). This group was composed of furfural, 2-acetyl-furan, and 2-furanmethanol. Furfural, possibly generated from 3-deoxypentosone via cyclization and dehydration, is known to emit strong caramel and fruit aromas [45]. Along with DDMP, furfural, which was outlined in the positive direction of PC1 in the PCA loading plot, was used to distinguish the VOC profiles of Indonesian, Malaysian, and Vietnamese NCS products (Figure 4). On the other hand, MRP furanones were composed of 4,5-dihydro-2-methyl-3(2H)-furanone, 2(5H)-furanone, pantolactone, and furaneol. 4,5-dihydro-2-methyl-3(2H)-furanone that may be generated during the Maillard reaction using thiamine as a precursor at pH 6–7 or higher [46]. Likewise, furaneol (4-hydroxy-2,5-dimethyl-3(2H)-furanone/HDMF), an important MRP substance owing to its low odor thresholds and attractive flavor properties, such as caramel-like notes, might be formed during the Maillard reaction from rhamnose [47]. Taken together, these results provide valuable information on the potent flavor characteristics of each NCS, which could vary for specific food and beverage applications. For instance, food products with a need for nutty and roasted aromas can use Japanese NCS products because they contain higher amounts of pyrazine, whereas those with a need for sweet caramel-like aromas should consider NCS products of ASEAN origin because of their higher concentrations of furans and pyranones.

### 3.3. MS-e-Nose Profiles

**Figure 5:**
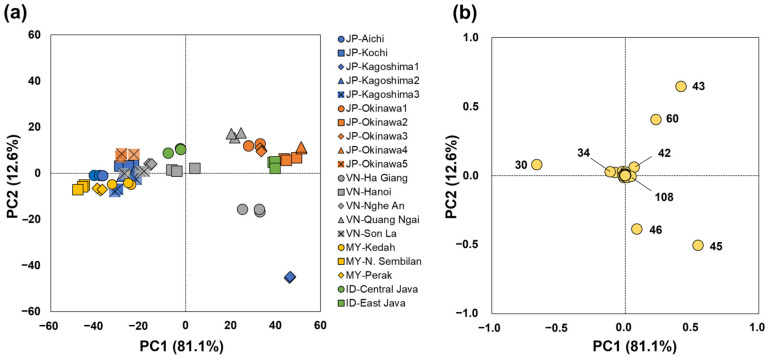
(**a**) PCA scores and (**b**) factor loadings of MS-e-nose profiles of NCS products from Japan and ASEAN countries.

**Figure 6:**
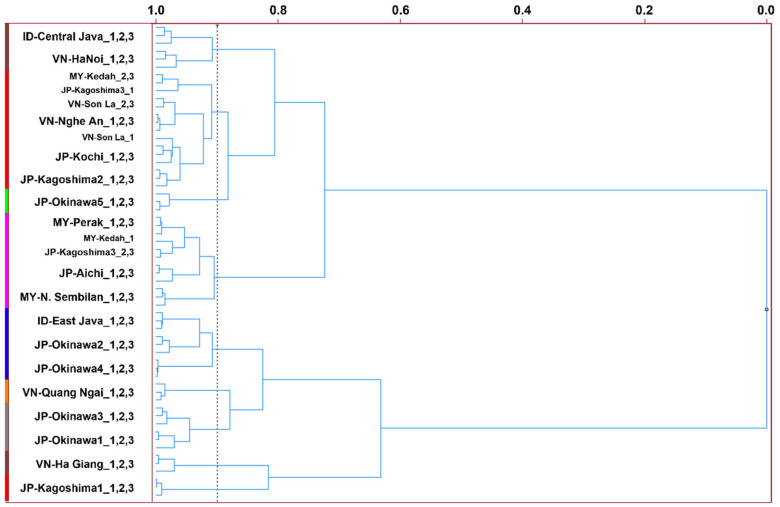
HCA dendrogram of MS-e-nose profiles of NCS products from Japan and ASEAN countries.

The MS-e-nose profiles show the captured ion masses of volatiles in NCS products from Japan and ASEAN countries as PCA and HCA plots, respectively (Figure 5 and Figure 6). The first two PC factors in the PCA contributed to 93.7% of the total variance, whereas some ion masses, such as *m/z* 30, 43, 45, 46, and 60, influenced the differentiation of the NCS products (Figure 5). The PCA score plot showed two major groups of NCSs (Figure 5a). The first group consists of NCS products from Malaysia (all three products); JP-Aichi, JP-Kochi, JP-Kagoshima2, JP-Kagoshima3, JP-Okinawa5, VN-Son La, VN-Nghe An, VN-Hanoi, and ID Central Java. These NCS products were in the negative direction of PC1, accounting for 81.1% of the variation, indicating comparable volatile profiles among products. The prominent chemical marker for the first NCS group was *m*/*z* 30, which was derived from an ion fragment of [C_2_H_6_]^+^ (Figure 5b) [48].

The second main group consisted of VN-Quang Ngai, JP-Okinawa1, JP-Okinawa2, JP-Okinawa3, and JP-Okinawa4 and was influenced by *m*/*z* 60, which was the quantifier of the predominant acetic acid, followed by m/z 43 from [C_3_H_7_]^+^ and [CH_3_C=O]^+^ fragments [49]. Ion masses at *m*/*z* 45 and 46, which were positively associated with PC1 and negatively associated with PC2, differentiated JP-Kagoshima1 and VN-Ha Giang from other NCS products. These ions could be derived from the corresponding MS fragment of ethanol and were potentially important “digital fingerprints” in MS-e-nose profiling for distinguishing various unrefined sugars made from different raw material sources [1]. Additionally, MS fragments from *m*/*z* 42 and 108, which were qualifier ions of the positional isomers 2,5-DMP and 2,6-DMP (and a small amount of 2,3-dimethyl pyrazine) [8], were plotted close to the center of the PCA loading plot. This indicates lower determining factors for NCS products from Japan and ASEAN countries. Nevertheless, ions at *m/z* 42 and 108 may contribute to the differentiation of the second main group of NCS products as well as JP-Kagoshima1 and VN-Ha Giang, which are outlined in the positive direction of PC1.

The PC scores obtained using the MS-e-nose technique indicate a specific aroma profile. The MS-e-nose aroma profile differentiated NCSs better than the composition of the identified VOCs. This can be attributed to the ability of the e-nose technique to capture additional loading factors from the overall volatiles emitted by the NCS products. The PCA produced using the composition of VOCs tended to accumulate in the center of the score plot, whereas the PCA produced using the MS-e-nose was dispersed throughout the PC1 score, particularly for Vietnamese, Malaysian, and Indonesian NCS (Figure 4 and Figure 5). Moreover, the MS-e-nose generated specific MS fragments from ethanol, acetic acid, and 2,5- and 2,6-dimethyl pyrazines, which are important chemical markers.

Nine statistical clusters of NCS products from Japan and ASEAN countries were observed using the HCA dendrogram, with a similarity index of 0.900 (Figure 6). We observed five clusters of mixed NCS products of different origins, suggesting similar intensity measurements for the MS fragments of the volatiles from those products and thus comparable volatile aroma profiles. The first cluster included all replicates of ID-Central Java and VN-Hanoi; the second cluster mainly consisted of MY-Kedah, VN-Son La, VN-Nghe An, JP-Kochi, and JP-Kagoshima2; and the fourth cluster included MY-Perak, JP-Kagoshima3, JP-Aichi, and MY-N. Sembilan. The fifth cluster included ID-East Java, JP-Okinawa2, and JP-Okinawa4 and the seventh cluster included JP-Okinawa1 and JP-Okinawa3. In contrast, four NCS products were exclusively identified as individual groups in the dendrogram: JP-Okinawa5 (third cluster), VN-Quang Ngai (sixth cluster), VN-Ha Giang (eighth cluster), and JP-Kagoshima1 (ninth cluster), indicating their sole and distinct volatile profiles.

HCA clustering distance also confirmed the differentiation profile of the PCA score plot (Figure 5a and Figure 6). For instance, VN-Ha Giang and JP-Kagoshima1, which were also plotted separately from the other NCS products in the PCA score plot, were remotely drawn in the dendrogram. In addition, although JP-Okinawa5 did not cluster with other NCS products in the HCA plot, it was drawn close to the dendrogram branch of MY-Kedah, VN-SonLa, JP-Kochi, and JP-Kagoshima2, with *m*/*z* 30 ion as the differentiating factor in the PCA loading plot (Figure 5b). Conversely, VN-Quang Ngai was closely drawn to the dendrogram branches of JP-Okinawa1, JP-Okinawa2, JP-Okinawa3, JP-Okinawa4, and ID-East Java in the PCA plot with *m*/*z* 43 and 60 as their chemical markers. These MS-based e-nose results detailed the differences between the volatile profiles of NCS products of Japanese and ASEAN origin. Moreover, the combined non-targeted volatile profiling technique and chemometric statistical data analysis provided important chemical markers in the form of a discriminative MS dataset for the further development of rapid measurement technology for flavor evaluation of NCSs or their derivative food and beverage products [1,8].

## 4. Conclusions

There was great variation in the mineral content of NCS products of Japanese and ASEAN origin, in which a distinction was observed domestically and between countries. The average mineral content was 962.87, 984.67, and 928.47 mg/100 g in Japanese, Malaysian, and Indonesian products, respectively, and was mainly composed of K, Ca, Mg, P, and Na, followed by small amounts of Fe, Zn, Mn, and Cu. In contrast, the average total mineral content in Vietnamese NCS products was 406.76 mg/100 g. Another exception was found in NCS from East Java, Indonesia, which contained much higher Na. A remarkable concentration distinction was also observed in VOCs, particularly in acetic acid and various preferred compounds derived from Maillard reactions, such as pyrazines, furans, and pyranones. Multivariate statistical analysis using PCA further revealed the association and differentiation of these NCS products based on their VOC composition. Moreover, the ion masses of the VOCs contributed to the construction of the PCA plot and HCA dendrogram of the MS-e-nose profiles, particularly at *m*/*z* 30, 43, 45, 46, and 60. The statistical plots of the MS-e-nose showed that some NCS products of Japanese and ASEAN origin had comparable volatile profiles. The distinctive minerals, VOCs, and volatile flavor profiles of each NCS might affect its overall quality characteristics, nutritional value, taste, and flavor potential. In broader applications, these analytical techniques could also be applied to a variety of flavor characteristic-driven food product developments, particularly for food and beverage products containing NCS. Taken together, the results of this study provide important information regarding the key nutritional and volatile flavor components of NCS products from Japan and ASEAN countries, and thus, could be used as a valuable basis for further preference and sensory studies for practical applications when they are used as sugar materials in food and beverage production. With the presentation of variations in minerals and VOCs among the evaluated NCS products, this study could exhibit the potency of compositional and MS-e-nose analyses in distinguishing the key quality attributes of these unrefined sugars; wider geographical areas across countries on different continents could be the next appealing study regarding NCS. However, the exploration of the functionalities of NCSs from different origins may become a topic for future research.

## Data Availability

The data are included in the article and supplementary materials.

## Supplementary Materials

The following supporting information can be downloaded at https://www.mdpi.com/article/10.3390/foods12071406/s1: Table S1. Concentrations and significant differences in minerals of non-centrifugal cane sugars of Japanese and ASEAN origin; Table S2. Volatile organic components of non-centrifugal cane sugars of Japanese and ASEAN origin.
